# Endoscopic observation of a lumen of a large hepatic cyst with aspiration and ethanol sclerotherapy: Case report and literature review

**DOI:** 10.1016/j.ijscr.2023.109001

**Published:** 2023-10-28

**Authors:** Shoryu Takayama, Takuya Banba, Takahumi Hyodo

**Affiliations:** a Department of Surgery, Haibara General Hospital, Hosoe 2887-1, Makinohara, Shizuoka 421-0421, Japan; b Department of Gastroenterology, Haibara General Hospital, Hosoe 2887-1, Makinohara, Shizuoka 421-0421, Japan

**Keywords:** A large hepatic cyst, Endoscopic evaluation, Deroofing

## Abstract

**Introduction and importance:**

A large hepatic cyst cause abdominal bloating and other symptoms. Surgical deroofing or ethanol sclerosis has been reported as the treatment options. We have treated patients surgically. However, an experience with postoperative bile leakage prompted us to reexamine our treatment options. It has been reported that the cause of bile leakage is the connection between the hepatic cyst and the bile duct. Therefore, we planned to observe the lumen of the hepatic cyst by endoscopy to evaluate the bile duct connection.

**Case presentation:**

An 82-year-old woman presented to our hospital for abdominal bloating. An abdominal computed tomography (CT) scan revealed a large hepatic cyst. Respiratory function was decreased due to diaphragmatic compression caused by the cyst. Endoscopic observation of the cyst was performed to evaluate the bile duct connection. There were no obvious abnormalities in her cyst. The patient was discharged 7 days after this procedure.

**Clinical discussion:**

Laparoscopic deroofing is recommended for the treatment of a large hepatic cyst when a patient can take surgery. However, deroofing has the potential for postoperative bile leakage. Careful consideration should be given to the treatment approach for each patient. Ethanol sclerotherapy has the potential for recurrence, but in this case, we confirmed the absence of bile duct connection. The ethanol sclerosis was effective, and there was no postoperative bile leakage.

**Conclusion:**

Endoscopic observation during puncture of the hepatic cyst allowed the evaluation of bile duct connection and search for malignant disease. Ethanol sclerotherapy was also effective.

## Highlights

• We report a case of endoscopic observation of the lumen of a large hepatic cyst.
• It allowed the evaluation of bile duct connection and search for malignant disease.

## 1 Introduction

This work has been reported as in line with the SCARE 2020 criteria [1]. Most simple hepatic cysts are asymptomatic and usually do not require treatment. However, large hepatic cysts can cause uncomfortable symptoms such as abdominal bloating, jaundice, portal hypertension, and leg edema due to compression of neighboring organs and the hepatic vasculature [2]. Therefore, patients with symptomatic giant liver cysts require treatment. Hepatic cysts are pathologically composed of an outer layer of fibrous tissue, lined by a cuboidal columnar epithelium that continually produces cystic fluid [3]. Therefore, simple puncture is not a curative procedure. Treatment methods reportedly include surgical deroofing and ethanol sclerotherapy after puncture [[4], [5], [6]]. Surgical treatment has been reported to be successful in up to 90 % of cases [7]. We have also performed laparoscopic deroofing because of the reported good surgical outcomes and the ability to evaluate for the presence of malignant disease. However, we experienced postoperative bile leakage after deroofing. It has been reported that bile ducts exist in the cyst wall and that bile leakage occurs as a result of damage to the bile ducts in the cyst wall. Indocyanine green fluorescence (ICG) navigation is reported to be effective in evaluating the bile duct migration [8]. There are also reports of cases in which the cyst wall and bile ducts had connection [9,10]. Various measures are needed to avoid postoperative bile leakage. There are few reports on effective methods of evaluating the connection between the hepatic cyst and the bile duct. In this case report, we report a case in which the bile duct connection was evaluated by endoscopic observation of the cyst under local anesthesia.

## 2 Case presentation

**Fig. 1:**
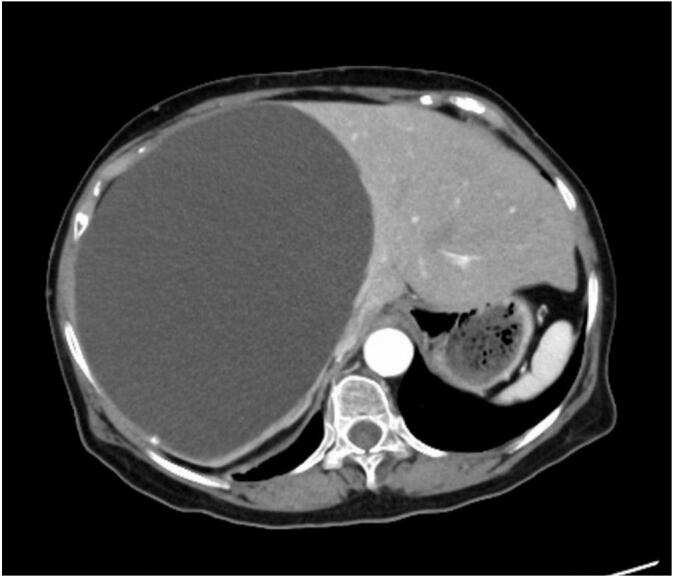
A large hepatic cyst. An 82-year-old woman came to our hospital because of abdominal distention. An abdominal computed tomography (CT) scan showed a large hepatic cyst.

**Fig. 2:**
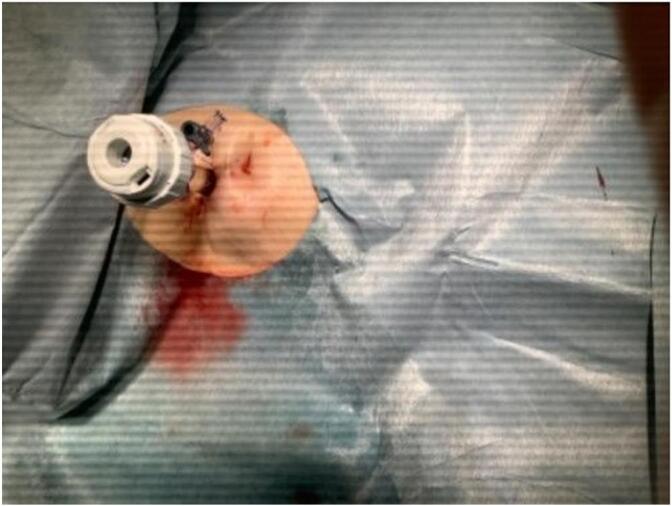
Port placement. To insert the port, the chest wall and cyst wall were fixed with nylon threads and adhered closely together. The port was inserted between the ribs under local anesthesia.

**Fig. 3:**
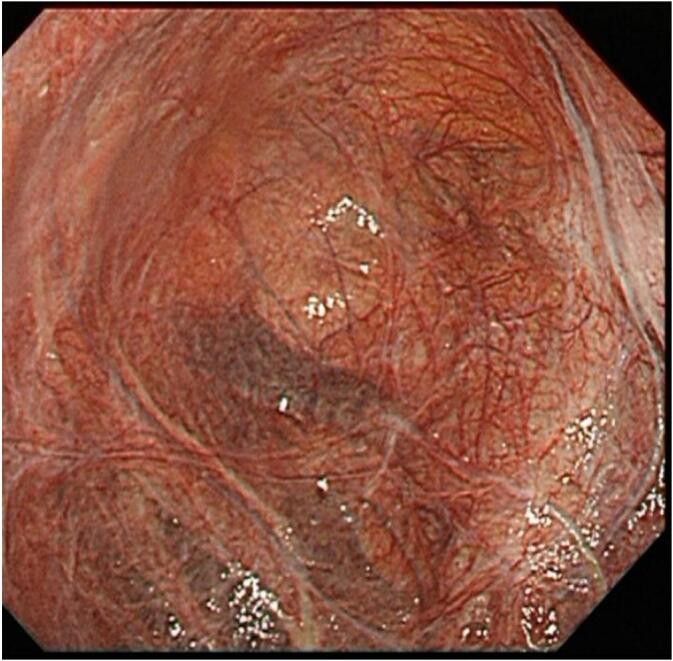
In the large hepatic cyst. We inserted an endoscope into the cyst through the port. The cystic fluid was completely aspirated and the cyst was dilated with carbon dioxide to fully observe the lumen. There were no malignant findings and no bile duct connection. On the lumen, some bile ducts exist.

An 82-year-old woman came to our hospital because of abdominal bloating. She had a history of cerebral infarction and hypertension and was being treated with clopidogrel. An abdominal computed tomography (CT) scan showed a large hepatic cyst (Fig. 1). Blood tests showed no abnormalities. Respiratory function was decreased due to diaphragmatic compression associated with the hepatic cyst. Because of her advanced age and decreased respiratory function, ethanol sclerotherapy was chosen instead of deroofing under general anesthesia. Since recurrence is a concern when there are connections between the hepatic cyst and the bile duct, we decided to perform ethanol sclerotherapy after observing the lumen of the cyst. If bile duct connection has been observed, we could have performed argon beam coagulation. If there have been any mass lesions that might be suspicious for malignant disease, we could have perfumed biopsy. To insert the port, the chest wall and cyst wall were fixed with nylon threads and adhered closely together. The port was inserted between the ribs under local anesthesia (Fig. 2). The cystic fluid was completely aspirated and the cyst was dilated with carbon dioxide to fully observe the lumen. There were no malignant findings and no bile duct connection (Fig. 3). A postoperative drain was placed and anhydrous ethanol was injected to adhere the cyst lumen. The drain was managed by applying negative pressure. The patient was discharged on postoperative day 7. At discharge, she said her symptoms of abdominal bloating had resolved and she could breathe more easily.

## 3 Discussion

Hepatic cysts are a common disease. Prevalence in the population has been reported to be 3–5 % in ultrasound series and as high as 15–18 % in CT series [11,12]. Asymptomatic liver cysts do not require treatment or follow-up [13]. Large liver cysts can cause uncomfortable symptoms such as abdominal bloating, jaundice, portal hypertension, and leg edema due to compression of neighboring organs and the hepatic vasculature [2]. Therefore, large liver cysts require treatment, but there is no standardized approach to treatment. Treatment methods are surgical deroofing and ethanol sclerotherapy by puncture [[4], [5], [6]]. Surgical treatment tends to be recommended due to its better outcomes and ease of management of treatment, including the search for malignant disease [7,14]. Compared to open surgery, laparoscopic surgery has no difference in treatment outcome and shortens hospital stay [15,16]. Therefore, laparoscopic deroofing is often the standard of care. However, postoperative bile leakage has been reported as a complication of deroofing. In some cases, bile ducts may run into the wall of the hepatic cyst. In such cases, the bile duct must be clipped, and it has been reported that ICG can be used to identify the bile ducts [17]. There are cases reported in which the cyst wall and bile ducts had connections [9,10]. In such cases, those bile ducts that have connections with the cyst wall are ligated and clipped. Since bile duct connections may be too small to be recognized, the effectiveness of argon beam treatment and deroofing with greater omentum flap has been reported [18,19]. We have also performed laparoscopic deroofing with greater omentum flap, but we have experienced postoperative bile leakage. Performing deroofing always has the possibility of complications of bile leakage. Hepatic cysts are benign diseases, and the benefits and harms of treatment should be considered on a patient-by-patient. In the case of this patient, her condition was not suitable for general anesthesia. Therefore, we first chose the ethanol sclerotherapy. To evaluate the presence of malignant disease and connections with the bile ducts, we observed the inside lumen of the hepatic cyst with dilatation by carbon dioxide. There were no obvious abnormalities. Ethanol sclerotherapy is a treatment to make adhere the lumen of the hepatic cyst. Therefore, we believe that sufficient adhesion cannot be achieved if cystic fluid remains in the cyst. Endoscopic aspiration was a good procedure to aspirate the cyst fluid completely. The insertion of a postoperative negative-pressure drain may have also facilitated the adhesion of the cyst wall. This technique is effective in searching for malignant disease, evaluating for bile duct connection, and ensuring that cystic fluid is aspirated. However, there are no reports of such a technique, and its safety needs further investigation. In addition, the follow-up is short, and its effectiveness as a treatment should be carefully studied.

## 4 Conclusion

An intra-cystic evaluation was performed endoscopically for the treatment of a large hepatic cyst. There was no obvious malignant disease or bile duct connection. Symptomatic large hepatic cysts require treatment, often treated by laparoscopic deroofing. Deroofing, however, has the potential for postoperative bile leakage. This technique allows preoperative evaluation of bile duct connection and the presence of malignant disease. This technique may be an option for the treatment of a large liver cyst.

## Consent

Written informed consent was obtained from the patient for publication of this case report and accompanying images. A copy of the written consent is available for review by the Editor-in-Chief of this journal on request.

## Provenance and peer review

Not commissioned, externally peer-reviewed.

## Ethical approval

Ethical approval for this study (No. 2023-3) was provided by the Ethical Committee of Haibara General Hospital, Shizuoka, Japan on 15th August 2023.

## Funding

Authors did not receive funding for this research.

## Guarantor

Shoryu Takayama.

## Research registration number

Not applicable.

## CRediT authorship contribution statement

All authors namely, Dr. Takahumi Hyodo, Dr. Takuya Banba and Dr. Shoryu Takayama were involved in the management of this patient. This manuscript has been drafted by all authors.

## Declaration of competing interest

The authors report no declarations of interest.
